# The Severe Acute Respiratory Syndrome Coronavirus-2 (SARS-CoV-2) Pandemic: Are Africa's Prevalence and Mortality Rates Relatively Low?

**DOI:** 10.1155/2022/3387784

**Published:** 2022-02-26

**Authors:** Solomon H. Mariam

**Affiliations:** Infectious Diseases Program, Aklilu Lemma Institute of Pathobiology, Addis Ababa University, Addis Ababa, Ethiopia

## Abstract

Severe acute respiratory syndrome coronavirus-2 (SARS-CoV-2), the cause of coronavirus disease 19 (COVID-19), has been rapidly spreading since December 2019, and within a few months, it turned out to be a global pandemic. The disease affects primarily the lungs, but its pathogenesis spreads to other organs as well. However, its mortality rates vary, and in the majority of infected people, there are no serious consequences. Many factors including advanced age, preexisting health conditions, and genetic predispositions are believed to exacerbate outcomes of COVID-19. The virus contains several structural proteins including the spike (S) protein with subunits for binding, fusion, and internalization into host cells following interaction with host cell receptors and proteases (ACE2 and TMPRSS2, respectively) to cause the subsequent pathology. Although the pandemic has spread into all countries, most of Africa is thought of as having relatively less prevalence and mortality. Several hypotheses have been forwarded as reasons for this and include warmer weather conditions, vaccination with BCG (i.e., trained immunity), and previous malaria infection. From genetics or metabolic points of view, it has been proposed that most African populations could be protected to some degree because they lack some genetic susceptibility risk factors or have low-level expression of allelic variants, such as ACE2 and TMPRSS2 that are thought to be involved in increased infection risk or disease severity. The frequency of occurrence of *α*-1 antitrypsin (an inhibitor of a tissue-degrading protease, thereby protecting target host tissues including the lung) deficiency is also reported to be low in most African populations. More recently, infections in Africa appear to be on the rise. In general, there are few studies on the epidemiology and pathogenesis of the disease in African contexts, and the overall costs and human life losses due to the pandemic in Africa will be determined by all factors and conditions interacting in complex ways.

## 1. Introduction

Since late 2019, severe acute respiratory syndrome coronavirus-2 (SARS-CoV-2), the cause of COVID-19, has plagued the world. At smaller scales, the world had previously experienced at least two respiratory viral epidemics since 2000. These were SARS-CoV-1, which emerged in China in 2003, and Middle East respiratory syndrome (MERS) in 2009-10. SARS-CoV-2 has proven that it can infect many millions and kill several millions.

Infected persons can have variable immune responses. Disease may vary from mild symptoms to severe pneumonia, respiratory failure, to death. Several factors including age, presence of comorbidities, environmental factors, and genetics contribute to the final outcome of the disease. Asymptomatic carriers can also spread the infection as they shed the virus [1].

SARS-CoV-2 contains structural proteins called M (membrane), E (envelope), and S (spike) proteins embedded in the viral envelope and a nucleocapsid (N) protein, which embeds the viral RNA in the inner core. The spike protein S binds through its receptor-binding domain (RBD) to its receptor angiotensin-converting enzyme 2 (ACE2) found on human cells [2, 3]. ACE2 is found on multiple organs including the lungs, heart, and kidneys. In the lungs, ACE2 is found on type II pneumocytes, the cells that are found on lung alveoli and that secrete surfactant needed for surface tension reduction and lung expansion during breathing. The bound viral S protein is then subsequently cleaved into two subunits: S1, the receptor-binding subunit, and S2, the membrane fusion subunit by the host cell protease TMPRSS2 resulting in fusion with the host cell membrane and entry into host cells, which initiates the subsequent pathology. SARS-CoV-2 invades larger surface area of the lung. It has a high affinity, even higher than that of SARS-CoV-1, for its receptor ACE2 [3]. SARS-CoV-1, MERS, and a bat coronavirus also use ACE2 as their receptor to enter human host cells [4, 5].

There can be several hallmarks of COVID-19. Excessive inflammatory responses involving multiple organs contribute to high morbidity and mortality. COVID-19 causes fluid accumulation in alveoli, causing reduced oxygen to reach blood. This leads to shortness of breath, tachypnea, and organ damage. Collectively, these are manifestations of acute respiratory distress syndrome (ARDS). It may also cause cardiovascular disease due to inflammatory cytokine surge [6, 7]. Unfavorable outcome in COVID-19 patients may also be caused by downregulation of ACE2, which otherwise has a protective role by reducing both vascular permeability and inflammation of the lungs and other tissues [8]. Formation of thrombus, which contributes to patient morbidity and mortality, is common in patients with the disease [9, 10]. COVID-19 also causes diffuse pulmonary intravascular coagulopathy in the lungs of COVID-19 patients [6].

## 2. Global Occurrence of SARS-CoV-2 Infections and COVID-19

Epidemics or pandemics are not new, but this pandemic is prominent in the magnitude of the economic and human life losses it is causing. In comparative terms, Africa seems to have slower rates of infectious cases and mortality. Supplementary Figure 1 (S.F.1.) depicts total cases and deaths, including per million populations, caused by the pandemic in countries from selected regions of five continents. The information in this table was obtained from Worldometers (accessed, January 6, 2022). From the data in S.F.1, it can be argued that not only Africa, but also Asia has the fewest number of COVID-19 deaths. Asia is the continent where the world's population giants are located. However, some Asian countries, such as Mongolia, Indonesia, Philippines, Nepal, and India, have been affected much more than other Asian countries, with deaths per million ranging from 345 in India to more than 600 in Mongolia. In Oceania, French Polynesia and Fiji have the highest rates of deaths per million, as the Worldometers data show. In S.F.1, it is of note that numerical values do not always convey the meaning or impact. Total cases and population sizes are also considered. For example, looking at S.F.1 D, it appears that both Seychelles and Germany have similar rates of deaths/million. However, the population sizes of the two countries are vastly different, being more than 80 million for Germany but about hundred thousand for Seychelles.

Furthermore, it is important to consider the age structures of populations of countries when evaluating the deaths/million. It is known that mortality due to the pandemic is highest in older age groups and, accordingly, some countries with pyramidal population age structures, which is especially true in most African countries, may appear to have lower death rates than countries having populations with higher median ages [11]. Higher death rates in infected people often happen to be associated with other comorbidities, which also often occur in older age groups.

## 3. The African Continent and SARS-CoV-2

Data seem to indicate the prevalence of infection or death rates due to COVID-19 are comparatively low in Africa. There have been several hypotheses forwarded, suggesting the factors responsible for the comparatively low infection and death rates in Africa. These include BCG vaccination, weather conditions considered less favorable for viral transmission in Africa, previous malaria infection, and genetic factors. In the following paragraphs, published information on the proposed hypotheses is discussed.

### 3.1. BCG Vaccination

**Figure 1:**
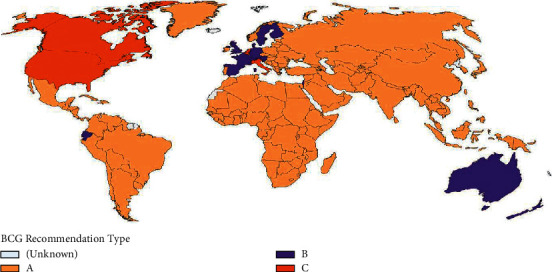
Map displaying BCG vaccination policy by country. A: the country currently has universal BCG vaccination program. B: the country used to recommend BCG vaccination for everyone, but currently it does not. C: the country never had universal BCG vaccination programs [12].

Africa as a whole adopts BCG vaccination programs to the new borne. Globally, the BCG vaccination policies of countries differ from those that never adopted it, such as France, Spain, and England, to those that once had the program but not anymore, such as Israel, to those that currently vaccinate but without a booster follow-up, such as the whole of Africa, Russia, and Middle and Far East countries [[12], Figure 1]. There are reports that BCG vaccination boosts immune response against COVID-19. Cross-reactive epitopes have been found to be shared between B and T cell epitopes of BCG and B and T cell epitopes of SARS-CoV-2 [13], with implications that these could lead to adaptive immune responses against SARS-CoV-2 in BCG-vaccinated individuals.

A study [14] compared mortality rates due to the current pandemic among the above three groups of countries. Their finding was that the median mortality rates per million ranged from 146.5 to 34 to 2.1, respectively, in the three groups of countries. Another early 2020 study [15] on the same similarly concluded that BCG vaccination history and death per million are negatively correlated, and the protection was long-lasting. These authors also argue that countries that only previously had BCG vaccination programs or that never implemented such programs had higher death rates than countries with current BCG vaccination programs, and the inverse correlations remained intact after controlling for confounding factors; that is, the inverse relationships were maintained in countries with similar socioeconomic conditions.

There are inconsistencies in efficacy of BCG vaccination in protecting COVID-19. For example, a following study [16] reported that the negative correlation described in the previous study [15] was no longer observed after April 2020. Kandeil et al. [17] reported that BCG and other childhood vaccines did not provide protective or neutralizing antibodies against SARS-CoV-2 in mice.

Some of the above discordant results could be attributable to several interacting factors, including (i) variations in study designs and possible bias also created with selection of study participants; (ii) the route of administration, i.e., whether it is oral, subcutaneous, intradermal, etc.; (iii) age at administration, previous exposure, coinfections [12, 18, 19]; (iv) probable differences in medical interventions, whether palliative or otherwise that can possibly impact on the outcome; (v) differences in the specific BCG strain used for immunization, i.e., whether BCG Pasteur, Russia, Danish, etc. were used. In connection to this specific BCG strain factor, a recent study [20] compared five licensed BCG vaccine strains with respect to their viability in culture and cytokine induction patterns and found that the BCG vaccines markedly differed in viability in culture and induction of chemokines and cytokines. BCG immunization data are also generally country-level, and these may not necessarily translate to individual patient-level data.

Further, some issues can be raised regarding the supposed protective effect of BCG against SARS-CoV-2. Countries such as New Zealand and Australia that do not currently have BCG vaccination programs have relatively low cases of infection and mortality. These countries initially had minimal infection rates because they are island nations and also instituted border closures and public-private public health measures. However, they have been recently hit by the more transmissible Delta and/or Omicron variants. Conversely, countries that have BCG vaccination programs, such as India and Brazil, have high rates of infection. It is also important to distinguish between two aspects of the supposed BCG effect: reductions in infection and mortality from COVID-19. These emerging BCG stories deserve serious consideration, and this pandemic has indeed given renewed interest for intensified clinical trials on BCG that are currently ongoing.

### 3.2. Temperature and Humidity

Low relative humidity (RH) and low temperature promote transmission, while high temperature and humidity within a certain range contribute to reduced transmission [21–25]. The seasonality of influenza viruses in temperate regions is well recognized [26]. Low AH and influenza usually co-occur during temperate Winters. Both viral stability and transmissibility are important. Low AH promotes influenza virus survival, transmission, and influenza-related deaths, because inhalation of dry air also impairs innate resistance and virus clearance [26–29]. In temperate Winters, low temperatures and both low and high RH permit survival of SARS-CoV-2 [25]. Besides, indoor air conditioning with low humidity and poor ventilation may also promote transmission [25].

Studies observed that SARS-CoV-2 could be inactivated by sunlight. High temperatures can cause decay of the virus and shorten its half-life, with reduced infectivity. Sunlight also inactivates SARS-CoV-2 on surfaces [30–32]. Indoor conditions can promote fomite and aerosol transmission. Decay of SARS-CoV-2 was accelerated when temperature and RH increase [32]. In outdoor conditions of intense sunlight, transmission of influenza and SARS-CoV-2 viruses would be reduced, depending on viral load and infectious dose [31–33]. Ultraviolet radiation causes viral inactivation and reduction of its reproduction numbers [33–36]. However, high infection rates have occurred at high temperatures, indicating the importance of combinations of infection control measures [35, 37, 38].

Other respiratory viruses can also be sensitive to high temperatures. Conversely, low temperatures favor transmission [24–26]. High temperature and humidity reduced transmission of SARS-CoV-1 and influenza virus [39, 40]. There is negative correlation between both AH and temperature and influenza viruses A and B [41]. Cold temperatures and low humidity favor transmission of the virus because of increased risk of respiratory tract infections [21, 42]. Similarly, TGEV and MHV viruses persisted on stainless steel for 28 days at 4°C, with some inactivation at 20°C [43] and rapid inactivation at 40°C. Another study [44] similarly described this bimodal nature of the RH effect and the nonlinear, monotonic effect of temperature on influenza virus. Similarly, in influenza A virus, survival was highest at RH values >100% or <50% [45].

A recent study [46] found that low temperatures favor increased RBD-ACE2 binding interaction and entry of SARS-CoV-2 leading to higher viral replication in the upper airways. That may provide a possible mechanistic explanation for a low temperature-enhanced infectivity of the virus and increased transmissibility during cold season [46]. Evolving lineages of SARS-CoV-2 with mutations in the Spike domain have been found that can also replicate at warmer temperatures of the lower airways, but if this would result in increased transmissibility is to be demonstrated as well [46, 47].

In Africa, humidity is high year-round and, along with high temperature, is considered to reduce survival and transmission of influenza virus [28]. However, sub-Saharan Africa has one of the highest influenza-associated deaths in the elderly [48], where fomites as well as close contacts probably promote more influenza transmission [49].

### 3.3. Malaria

Malaria is the other factor regarded as a possible reason for the low rate of infection in Africa. According to World Health Organization reports, the African region is characterized by a high prevalence of malaria (∼150.9 million in 2018), with the dominant infection caused by *Plasmodium falciparum* (99%, compared to ∼0.7% of *P*. *vivax* in 2018). The mechanism of protection is posited to be cross-reactivity between SARS-CoV-2 and *Plasmodium* antigens. Vanroye et al. [50] found similar cross-reactivity of antibodies against SARS-CoV-2 with not only *P*. *falciparum* but also other species of current and recent *Plasmodium* infections as well. An Italian study [51] also found that regions of southern Italy with the lowest number of COVID-19 were those that had the highest incidence of malaria several decades ago. But the mechanism for this inverse correlation between the incidence of malaria decades ago and the current pandemic is still speculative. Similar immunodominant epitopes were found between *P*. *falciparum* and SARS-CoV-2 antigens [52]. This means cross-immunogenic reactivity or cross-protection between *P. falciparum* and SARS-CoV-2 antigens, TRAP, and SSP-2, respectively, suggesting that previously malaria-infected people have antibodies developed due to established memory against *P*. *falciparum*, which react with SARS-CoV-2 antigens, and this accounts for the low viral infection rate in malaria-endemic regions of Africa. This phenomenon is also thought to develop in hosts previously exposed to other bacterial or viral infections. It is referred to as cross immunity [53]. However, it is important to note that there are non-African countries with low numbers of SARS-CoV-2 infection and that were free of malaria. Thus, malaria can be ruled out as having any role in the epidemiology of the pandemic in those countries. Finally, how many of those with SARS-CoV-2 infections in Africa had malaria and how many of those were spared need to be tested, after adjusting for any comorbidities.

Chloroquine and hydroxychloroquine are drugs that have been used for treatment of malaria. These drugs had been proposed as possible prophylactic or therapeutic drugs against SARS-CoV-2-based on previous knowledge that these drugs showed inhibitory effects in vitro against some common viruses infectious to humans. However, several recent studies or systematic reviews concluded that the drugs have no therapeutic value in preventing or treating SARS-CoV-2 infections in humans, probably because tolerable therapeutic serum concentrations cannot be achieved in vivo [54–56].

### 3.4. Vitamin D Levels

Several studies indicated that vitamin D supplementation could benefit SARS-CoV-2 control measures [57–60]. Vitamin D deficiency increased risk to severe SARS-CoV-2 infection outcomes and admission to ICUs in persons with vitamin D deficiency. Conversely, the need for admission to ICU was reduced in patients after administration of calcifediol, a vitamin D analog that helps in metabolism of calcium [61–63]. Other reports suggested that vitamin D levels in African populations are generally low [61, 64, 65]. Therefore, this phenomenon does not favor the idea of lower rate of SARS-CoV-2 infection prevalence in Africa attributable to vitamin D, but possible factors other than vitamin D contribute to the protection. It may also be that its benefit is reduced due to presence of comorbidities. Controlled trials are needed to clarify this ambiguity. The general consensus is to give vitamin D supplementation to vitamin D-deficient patients, given the indirect evidence of its benefit for optimal immune function and the relative safety of its administration [66, 67].

### 3.5. Genetic/Metabolic Factors

#### 3.5.1. Chromosome 3

A genetic basis for susceptibility to SARS-CoV-2 infection has been put forward. Two studies [68, 69] found that carriage of a 6-gene cluster on chromosome 3 is a risk factor and associated with the severity of SARS-CoV-2 infection and respiratory failure. This gene cluster was inherited from Neanderthals (ancient hominids) [69]. These gene clusters are almost completely absent from Africa, consistent with the idea that gene flow from Neanderthals into African populations was limited [69]. A region of chromosome 3, i.e., 3p21.31, containing 12 protein-coding genes may be involved in SARS-CoV-2 pathogenesis when it carries single nucleotide polymorphisms (SNPs) in its variant form. This variant enhances complement fixation and infiltration of lung tissues with monocytes and macrophages in infected cases (a hallmark of severe disease), resulting in severe inflammatory responses [70, 71].

#### 3.5.2. Gene Polymorphisms

A German study [72] analyzed SNP variants of TMPRSS2, which included rs2070788, rs383510, and rs12329760. Of these, only rs383510 was associated with increased risk (∼2-fold) of infection or disease severity. However, allele variants of both rs2070788 and rs383510 were shown to be significantly associated with susceptibility to infection by H1N1 and H7N9 influenza viruses in Chinese patients [73]. It appears that these variants are expressed, alternatively or to variable degrees, in different ethnic populations. The allele frequencies of these variants are comparatively lower in Africans than in Europeans and East Asians [72].

The expression level of ACE2 and TMPRSS2, or allele frequencies of their variants, was found to be significantly lower in African people of both genders than in Europeans and East and South Asians [74]. Another study [75] found strong correlation between case fatality rate and SNPs in several relevant genes including other polymorphisms in TMPRSS2 and ACE2. An inhibitor of TMPRSS2 that has been found to block entry of SARS-CoV-2 might offer a therapeutic option [2].

#### 3.5.3. Alpha (*α*)-1 Antitrypsin

Studies implicate *α*-1 antitrypsin deficiency (a recessive, heritable disease) in exacerbating coronavirus disease. *α*-1 antitrypsin is produced by hepatocytes and secreted into the blood. It acts as a circulating serine protease inhibitor, and its principal target is the protease neutrophil elastase, which is released by damaged neutrophils [76]. *α*-1 antitrypsin protects the lung and other tissues containing elastin. The normal allele is PiM. Many genetic variants (alleles) of *α*-1 antitrypsin cause *α*-1 antitrypsin deficiency. The most common variant alleles are PiS and PiZ. Most (96%) of *α*-1 antitrypsin deficiency is linked to the PiZZ type, causing very low *α*-1 antitrypsin production, while the other variants produce low-intermediate levels [76–78]. The deficiency can also make people susceptible to developing asthma, bronchitis, chronic obstructive pulmonary disease, etc. The number of deaths due to the current pandemic and prevalence of *α*-1 antitrypsin deficiency are strongly positively correlated in many countries [78, 79].

Recent studies on the global epidemiology of *α*-1 antitrypsin deficiency revealed that Africa as a whole has the lowest rates of these deficiency alleles. In North Africa, several countries were reported to have no PiS or PiZ prevalence. Several central and south African countries have PiS frequencies ranging from 1.8 to 63 per 1,000 population. In other African countries, the predominant allele was generally the milder form, and the PIZ frequency is very low or absent [76, 77]. Some regions of eastern and western Africa were found to have moderate frequencies of PIZ allele [80]. In European countries, both alleles are found at variable frequencies, e.g., 2.0 in Greece to a high of 76 per 1,000 population in France [77]. Countries in Europe with the highest rates of SARS-CoV-2 disease and death were also those with the highest rates of alleles causing *α*-1 antitrypsin deficiency, as previously shown [81]. In North and Central American countries, both alleles were found in all countries, but the PiS allele is predominant, being 23 to 45 per 1,000 population. In Asia, the prevalence of the two alleles is generally low to medium, ranging from 1.0 to 31 for PiS and 0.2 to 15.0 for PiZ. Of note, Indonesia, Mongolia, and Nepal have zero prevalence of both alleles, while China has a prevalence of 1.0 and 0.0 per 1,000 population for PiS and PiZ, respectively. In Australia and New Zealand, the prevalence of PiS is 42 and 12, while the prevalence of PiZ is 33 and 26, respectively, per 1,000 population [76, 77].

#### 3.5.4. C-Reactive Protein (CRP)

Elevated levels of CRP have also been implicated as a marker of COVID-19 disease severity in patients. The CRP is produced in the liver in response to increased inflammatory cytokines such as interleukin-6 (IL-6), and levels of these two biomarkers are positively correlated [82]. CRP release is associated with conditions such as hypertension, obesity, cardiovascular disease, and ARDS, or infections with influenza viruses such as H1N1, MERS, and SARS-CoV-2 [83–85]. Recent studies indicate that CRP levels in COVID-19 patients who were severely ill or died were significantly higher than those in surviving patients [86, 87]. Elevated CRP levels during the first 2-3 days of hospital admission can be used prospectively as predictors to identify those patients who would deteriorate to respiratory failure and require intensive care, including intubation [82, 88].

Studies of CRP levels in association with COVID-19 in Africa appear to be rare, but the metabolic marker may not be rare. Some reports indicated that CRP levels were associated with adverse outcomes in patients [89–91]. In general, CRP levels are high in many African countries or in African Americans, especially in association with diabetes, hypertension, or infectious diseases [92].

#### 3.5.5. Blood Type

ABO blood groups have been suggested to be associated with outcomes of infection with other viruses or bacteria, e.g., type O being more susceptible to Norwalk virus, *Helicobacter pylori* or SARS- CoV-1 [93–95]. Some reports indicate that blood type A is more susceptible to SARS-CoV-2 infection. These suggest that the RBD of SARS-CoV-2 preferentially binds to type A antigens specifically located on human lung epithelial cells [96]. Still, reported results regarding blood type susceptibility to SARS-CoV-2 are conflicting—some reporting type A, though more susceptible, do not need intubation, and type O has the lowest risk [97]. In Africans, type A is reported to be at low frequency, while type O occurs at the highest frequency [98].

Prevalence of disease relative to blood type should be adjusted to known prevalence of blood types in the general population since proportions of blood types in patients may not be representative of the actual proportions in the general populations. Similarly, blood type distributions show variability in different races. Furthermore, presence of other comorbidities may influence or override blood type susceptibility. Most studies also suffer from insufficient sample sizes. Studies based on large samples indicate no associations of blood types to infection or COVID-19 severity, or only speculate that type O may be associated with a lower risk, while type A may be associated with a higher risk [99, 100]. This blood type-COVID-19 interaction also awaits further clarification.

## 4. Role of Geographic Location, Race, and Ethnicity in the Spread of the Global Pandemic

Studies show that this pandemic hits differentially at continental, country, and at subcountry levels [101]. Studies in the United States indicated that the distribution of the pandemic exhibited disparities based on geography, resulting in differential exposure risk, testing rates, and access to health services. These studies also show that the disparities based on geography intermingle with other factors such as race, ethnicity, and age to contribute to the disparities. Zalla et al. [102] argue that geography in relation to race, including where one lives and works, should be considered because race- and ethnicity-based desegregation have created the geographical landscapes where people have increased risks (e.g., poor housing, sanitation, working conditions, and access to health care) to be exposed, infected, and possibly die from the infection. In a retrospective cohort study of more than 19,000 patients in the United States, mortality—both crude and after adjustment for demographic factors and comorbidities—was found to be slightly higher in African-American patients than White patients [103]. Others found similar results and showed that once infected with SARS-CoV-2, African-American patients were at significantly higher odds to require hospitalization and to die than White patients [104–106]. Furthermore, African-Americans are reported to have higher rates of comorbidities such as diabetes and hypertension. But, one study found adjusted mortality risk to be lower for African-Americans [107]. Hospitalization and mortality rates in Hispanics were reported to be mostly similar to those of African-Americans, but there are some conflicting results. A more recent study regarding vaccination further indicated that priority should be given for all high-risk geographic areas without regard to age, because these people have lower median ages and therefore are more likely to be infected at younger ages and have higher risk of death from COVID-19 [108].

In a COVID-19 study in England [109], patients from minority groups had higher odds of adverse outcomes to test positive, require hospitalization, and die than White patients. The odds were especially much higher in these groups to require admission to the intensive care unit even after adjusting for age, gender, economic conditions and comorbidities.

In sub-Saharan Africa, it is the same race. Therefore, race would not be considered as major factor contributing to disparities in the pandemic distribution although there would be genetic differences. Still, the pandemic has generated variable effects in African countries. Regarding ethnicity, there are many ethnic groups in most African countries [110]. There appear to be no studies on possible interactions between ethnicity and the pandemic in Africa. There are several studies of the effect of the pandemic on people of African descent elsewhere (e.g., African-Americans), but it would be invalid to make any deductions from the results of such studies to Africans living in Africa. Africans and African-Americans live and work in geographically different areas and are also likely to differ in diet, life styles, etc. Recent studies, however, indicate that obesity, diabetes, and hypertension are increasing in Africa or are projected to increase [111–113].

Another aspect of the geographic distribution of COVID-19 concerns the different SARS-CoV-2 variants that have different temporal and spatial origins globally. Thus, the Alpha variant was initially documented in the United Kingdom in September 2020, while the Beta variant was initially recognized in South Africa in May 2020. The Gamma and Delta variants were first documented in Brazil and India, respectively, in October-November 2020. Lately, the Omicron variant emerged in multiple countries in November 2021 [114]. The variations occur especially in the Spike protein and may have several implications including adaptive evolution, speciation, transmission, or immune evasion [115].

## 5. The African “Paradox”

The rates of cases and COVID-19 fatalities in Africa are believed to be low. Some call this paradoxical, as if Africa should have been hit worse by this pandemic. There are some suggested reasons for the “low” rates. One is that many African countries responded timely to control the pandemic by travel restrictions, limited international connections, school closures, etc. The other is the age factor. Africa has the youngest population globally. This seems reasonable, since older age groups are the worst affected. For example, in Chile, publicly available data showed that senior citizens over 70 years of age had the highest case fatality rates even though younger age groups had the highest number of cases [116]. In New York, infection fatality risk was more than 100-fold higher in people ≥75 years during the first wave [117]. Similarly, a nationwide study in Spain showed infection fatality risk increased sharply in people over 50 years [118]. Such increasing mortality rates in older age groups were also observed in seven other economically advanced countries [119].

## 6. Is South Africa an Outlier?

South Africa is regarded as an outlier in Africa by many. In South Africa, several SARS-CoV-2 lineages with unique mutations were identified during the first wave, and most of these lineages had spread widely before lockdown and travel restrictions were imposed [120]. Rapid transmission also continued in South Africa from imported cases down to the community even during lockdown [121]. South Africa experienced very high mortality during both the first and second waves, but most African countries also had higher second-wave deaths [122]. In a South African study, human immunodeficiency virus and tuberculosis infections were associated with a 2-fold increase COVID-19 mortality [123]. South Africa has the highest number of total deaths, but it is exceeded by Tunisia in the number of deaths/million people. Botswana, Seychelles, and Libya also have the highest numbers of COVID-19 deaths per million in Africa (but less than South Africa). Finally, it is of note that testing and reporting capacities differ among countries.

## 7. Conclusions

It is important to know the role(s) of malaria, BCG vaccination, BCG strain type, blood type, age group, genetic background, comorbidities, etc., and their interactions when one is superimposed over the others. Effects of sunlight, high temperatures, and UV in reducing survival and transmissibility of the virus, even if positive, may be masked by other risk factors. It may also be too early to speak of low rates of the infection or mortality in Africa because the pandemic is still spreading. The available evidences call for more randomized, controlled studies that take into account all possible variables. The suggested protective effects by BCG, weather, etc. cannot substitute other preventive measures such as face masks, ventilation, and physical distances; fortunately all of them are relatively inexpensive and effective when combined. The microbiome may also play a role in these complex interactions and needs to be explored. Furthermore, the changing variants of SARS-CoV-2 might bring in different responses in African or other populations. Another point is that most of the studies in the published literature were not done in African context.

In a continent where only a small percentage of the population has been tested due to limited testing capacity, perhaps many of those who are infected but do not show any symptoms, or show only mild symptoms, harbor the true burden of the infection in Africa. Low rates of testing and underreporting lead to underestimation of the true cases, mortality rates, and contribute to further transmissions. Seroprevalence surveys in otherwise healthy people have also shown that these exceed those reported after molecular tests were conducted. Some symptoms that overlap among influenza, the common cold, pneumonia, and SARS-CoV-2 infection could mislead people to consider such symptoms as usual. Likewise, there can be COVID-19 deaths that are mistakenly attributed for other causes. Rates of infection are increasing from time to time in some African countries, suggesting that it is still early to speak of Africa as having low rates of infection and mortality from COVID-19.

## Data Availability

All data have been included in the manuscript and supplementary material.

## Supplementary Materials

Supplementary Materials Supplementary Figure 1. Reported COVID-19 cases and deaths per million populations for selected countries from 5 continents can be found online.
