# Supplementary material for: The Severe Acute Respiratory Syndrome Coronavirus-2 (SARS-CoV-2) Pandemic: Are Africa's Prevalence and Mortality Rates Relatively Low?
Source: Adv Virol. 2022 Feb 26;2022:3387784. doi: 10.1155/2022/3387784 (PMC8898136; doi:10.1155/2022/3387784)
Supplement: Supplementary Materials — Supplementary Figure 1. Reported COVID-19 cases and deaths per million populations for selected countries from 5 continents can be found online. [file 3387784.f1.docx]

A

B

C

D

EF Supplementary Figure 1. Reported COVID-19 cases and deaths per million populations for selected countries from 5 continents. A: total cases, B: total case/million, C; total deaths, D: total deaths/million, E; tests/million, F; population. Source: Worldometers (accessed on, January 6, 2022).
